# High-Affinity Ratiometric Fluorescence Probe Based on 6-Amino-2,2′-Bipyridine Scaffold for Endogenous Zn^2+^ and Its Application to Living Cells

**DOI:** 10.3390/molecules27041287

**Published:** 2022-02-14

**Authors:** Masayori Hagimori, Fumiko Hara, Naoko Mizuyama, Takeshi Fujino, Hideo Saji, Takahiro Mukai

**Affiliations:** 1 Laboratory of Analytical Chemistry, Faculty of Pharmaceutical Sciences, Mukogawa Women’s University, 11-68 Koshien Kyubancho, Nishinomiya 663-8179, Japan; fhara@mukogawa-u.ac.jp; 2 Division of Medical Innovation, Translational Research Center for Medical Innovation, 1-5-4 Minatojima-minamimachi, Chuo-ku, Kobe 650-0047, Japan; hagimori@tri-kobe.org; 3 Graduate School of Science and Engineering, Saitama University, 255 Shimo-Okubo, Sakura-ku, Saitama 338-8570, Japan; fujino@mail.saitama-u.ac.jp; 4 Graduate School of Pharmaceutical Sciences, Kyoto University, 46-29 Yoshida-Shimoadachi-cho, Sakyo-ku, Kyoto 606-8501, Japan; hsaji@pharm.kyoto-u.ac.jp; 5 Laboratory of Biophysical Chemistry, Kobe Pharmaceutical University, 4-19-1 Motoyamakita Machi, Higashinada-ku, Kobe 658-8558, Japan; tmukai@kobepharma-u.ac.jp

**Keywords:** Zn^2^^+^, ratiometric fluorescence probe, 6-amino-2,2′-bipyridine, small-molecular-weight, cellular imaging

## Abstract

Zinc is an essential trace element involved in many biological activities; however, its functions are not fully understood. To elucidate the role of endogenous labile Zn^2+^, we developed a novel ratiometric fluorescence probe, 5-(4-methoxyphenyl)-4-(methylsulfanyl)-[2,2′-bipyridin]-6-amine (**6** (**rBpyZ**)) based on the 6-amino-2,2′-bipyridine scaffold, which acts as both the chelating agent for Zn^2+^ and the fluorescent moiety. The methoxy group acted as an electron donor, enabling the intramolecular charge transfer state of **6** (**rBpyZ**), and a ratiometric fluorescence response consisting of a decrease at the emission wavelength of 438 nm and a corresponding increase at the emission wavelength of 465 nm was observed. The ratiometric probe **6** (**rBpyZ**) exhibited a nanomolar-level dissociation constant (*K*_d_ = 0.77 nM), a large Stokes shift (139 nm), and an excellent detection limit (0.10 nM) under physiological conditions. Moreover, fluorescence imaging using A549 human lung adenocarcinoma cells revealed that **6** (**rBpyZ**) had good cell membrane permeability and could clearly visualize endogenous labile Zn^2+^. These results suggest that the ratiometric fluorescence probe **6** (**rBpyZ**) has considerable potential as a valuable tool for understanding the role of Zn^2+^ in living systems.

## 1. Introduction

Zinc is an essential trace element for living organisms and plays pivotal roles in many biological processes, such as gene expression, apoptosis, enzyme regulation, and neurotransmission [1,2,3,4]. Most zinc is tightly bound to proteins and is involved in structural and catalytic functions [2,3]. In contrast, labile (free) zinc exits at low concentrations (picomolar to nanomolar) and is reported to play important roles in signaling, cell proliferation, and differentiation [4,5,6]. The amount of labile zinc present is regulated by zinc homeostasis; however, it has been suggested that zinc deficiency induced by insufficient dietary intake, poor absorption, and heredity disorders is related to hypogeusia, growth retardation, and carcinogenesis [7,8,9]. Therefore, to elucidate the details of the function and behavior of zinc in cells and tissues, a reliable method is urgently required.

Biological imaging using fluorescence probes is a robust method that enables the sensitive, real-time, and noninvasive detection of target molecules. Organic molecule-based fluorescence probes are gaining importance as simple cost-effective probes with good biocompatibility [10,11]. Their properties, including target binding ability and fluorescence response, are relatively easy to optimize by modifying their molecular structure and substituents [10,11]. As zinc has a closed shell structure (3d^10^4s^0^) and cannot be directly analyzed using conventional methods, such as ultraviolet/visible (UV/vis) and nuclear magnetic resonance (NMR) spectroscopies, the development of organic molecule-based fluorescence probes for zinc has attracted attention [12,13]. A variety of fluorescence Zn^2+^ probes based on fluorophore platforms, such as quinoline, fluorescein, cyanine, and biheteroaryl have been reported, some of which have contributed to the understanding of the biological function and behavior of zinc [12,13,14,15,16]. However, even with these probes, the detailed biological functions of zinc are yet to be fully understood. Therefore, the development of novel fluorescence Zn^2+^ probes that demonstrate higher affinity toward Zn^2+^ as well as increased solubility and cell membrane permeability than the currently available probes is essential. Previously, we reported a 6-amino-2,2′-bipyridine-based fluorescence Zn^2+^ probe **3**, in which the core structure acted as both the chelating agent for Zn^2+^ and the fluorescent moiety. The resulting probe had a small molecular weight, making it highly soluble and cell membrane-permeant [17]. This probe also achieved a nanomolar-range dissociation constant toward Zn^2+^ by controlling intramolecular amino-imino tautomerization; however, the high background fluorescence of the probe was a significant drawback for detecting endogenous Zn^2+^ [17]. The use of ratiometric probes enables highly sensitive measurements through self-calibration at different emission wavelengths in the presence and absence of analyte [18,19]. Generally, the ratiometric property is based on forming an intramolecular charge transfer (ICT) state. This is generally achieved by complex electron interaction between an electron donor and an electron acceptor within molecules [18]. In this manner, the ratiometric probe can minimize the effect of its background fluorescence, which allows for the accurate detection of analytes, including Zn^2+^.

We report a novel ratiometric Zn^2+^ probe based on the 6-amino-2,2′-bipyridine scaffold. The introduction of an electron-donating or electron-withdrawing group significantly affected the fluorescence property of the 6-amino-2,2′-bipyridine-based fluorescence probe, and 5-(4-methoxyphenyl)-4-(methylsulfanyl)-[2,2′-bipyridin]-6-amine, **6** (**rBpyZ**), exhibited a favorable ratiometric property and high binding affinity toward Zn^2+^. In this study, we demonstrate the design, synthesis, and fluorescence properties of **6** (**rBpyZ**) and evaluate its usefulness in detecting endogenous Zn^2+^ through fluorescence imaging using A549 human lung adenocarcinoma cells.

## 2. Results and Discussion

### 2.1. Synthesis of 6-Amino-2,2′-Bipyridine-Based Fluorescence Compounds

**Scheme 1:**
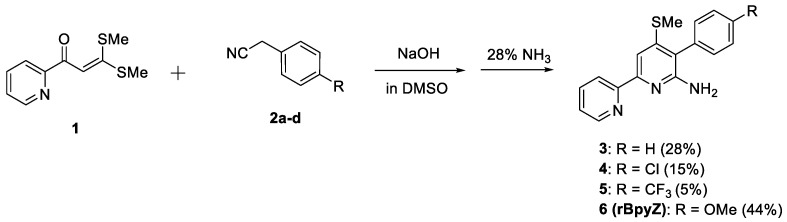
Preparation of 6-amino-2,2′-bipyridine-based compounds **3**–**6** (**rBpyZ**).

The 6-Amino-2,2′-bipyridine-based compounds **4–6** were synthesized using a one-pot synthesis method we developed for **3** [17], involving the reaction of 3,3-bis(methylsulfanyl)-1-(pyridin-2-yl)prop-2-en-1-one (**1**) with phenyl acetonitriles (**2b–d**) in the presence of sodium hydroxide, followed by treatment with an ammonia solution (Scheme 1). Compared to the yield of the unsubstituted compound **3** (28%), those of **4** and **5,** which bear electron-withdrawing groups (Cl and CF_3_) on the allyl group at position 5 of the pyridine ring, decreased to 15% and 5%, respectively. In contrast, the yield of **6** (**rBpyZ**), which bears an electron-donating group (OMe), increased to 44%. As these reactions are initiated by the nucleophilic attack of phenyl acetonitriles on **1**, it is likely that the electron density of the substituent in phenyl acetonitriles has a significant effect on its nucleophilicity, resulting in the difference in yields.

### 2.2. Spectroscopic Studies

**Figure 1:**
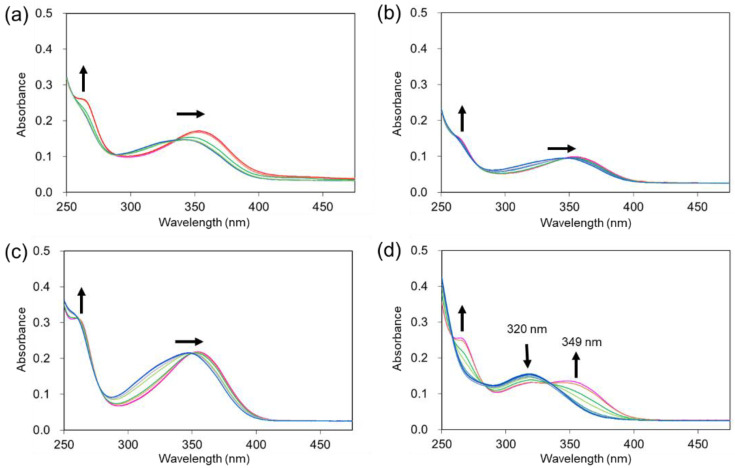
UV/Vis absorption spectra of 10 µM of (**a**) **3**, (**b**) **4**, (**c**) **5**, and (**d**) **6** (**rBpyZ**), in HEPES buffer (100 mM, 50% EtOH, pH = 7.4), The arrows indicate the direction in which the spectra shift when in the presence of Zn^2+^ as its perchlorate salt (0–1000 µM).

We analyzed the UV/Vis absorption and fluorescence properties of **4**–**6** (**rBpyZ**) in HEPES buffer (100 mM, 50% EtOH, pH = 7.4) and compared them with those of compound **3**. As shown in Figure 1, the absorption peaks at wavelengths 260 and 345 nm of free **3** gradually increased with the addition of Zn^2+^, accompanied by a small bathochromic shift of 5–7 nm (Figure 1a). Compounds **4** and **5** also exhibited absorption maxima at 260 and 345 nm, and similar changes to **3** were observed after adding Zn^2+^ (Figure 1b,c). In compound **6** (**rBpyZ**), the absorption peak at the longer wavelength exhibited a hypsochromic shift of 20 nm compared to that of **3**, and a new peak appeared at 349 nm with the addition of Zn^2+^ (Figure 1d).

**Figure 2:**
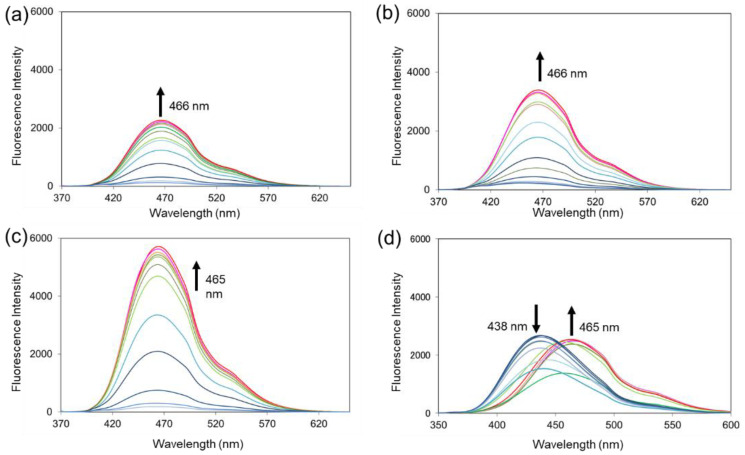
Fluorescence spectra of (**a**) **3** (1 μM, Ex_max_ = 359 nm); (**b**) **4** (1 μM, Ex_max_ = 360 nm); (**c**) **5** (1 μM, Ex_max_ = 359 nm); (**d**) **6** (**rBpyZ**) (1 μM, Ex_max_ = 326 nm) upon the addition of Zn^2+^ as the perchlorate salt (0–100 μM) in HEPES buffer (100 mM, 50% EtOH, pH = 7.4). Ex_max_ = excitation wavelength.

Figure 2 shows the fluorescence titration spectra with Zn^2+^. Similar to the spectrum of **3**, the fluorescence intensities of **4** and **5** gradually increased in a Zn^2+^ concentration-dependent manner, and the maximum emission wavelength of **4** and **5** exhibited small bathochromic shifts of 14 and 5 nm, respectively. The fluorescence intensities of **4** and **5** were stronger than that of **3**, being enhanced 10- and 18-fold, respectively. 

**Figure 3:**
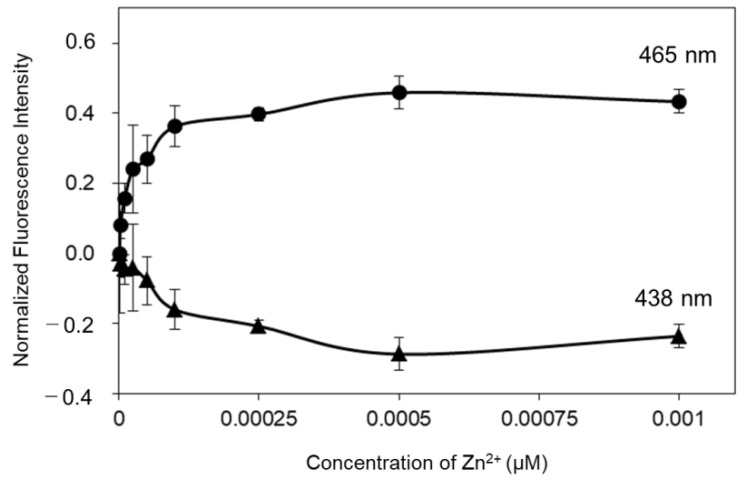
Normalized fluorescence intensity of **6** (**rBpyZ**) at 438 nm (▲) and 465 nm (●).

In contrast, a significant ratiometric response to Zn^2+^ was observed in compound **6** (**rBpyZ**) (Figure 2d). In the absence of Zn^2+^, **6** (**rBpyZ**) exhibited an emission peak at 438 nm, which was blue-shifted by 30 nm compared to those of compounds **4** and **5**. Upon adding Zn^2+^, the emission peak at 438 nm gradually decreased, and a new emission peak at 465 nm concomitantly appeared (Figure 3). In addition, **6** (**rBpyZ**) showed a significantly larger Stokes shift (139 nm), which enabled sensitive detection without interference from the excitation light wavelength.

These results indicated that a chelation-enhanced fluorescence (CHEF) effect occurred with the addition of Zn^2+^, and the simple replacement of substituent influenced the fluorescence emission profile. The analysis of all compounds revealed that the complex formed between the compound and Zn^2+^ possessed a 1:1 stoichiometry (Figure S1, Supplementary Materials). From the fluorescence titration data, the dissociation constants (*K*_d_) of **4**–**6** (**rBpyZ**) were calculated to be at a nanomolar level, comparable to that of compound **3** (2.2 nM) (Table 1; Figure S2).

**Figure 4:**
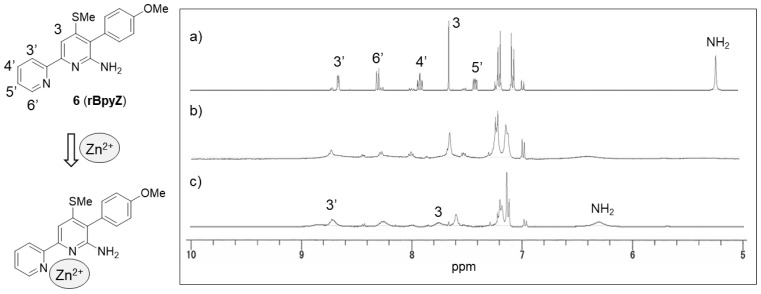
^1^H-NMR spectra of (**a**) **6** (**rBpyZ**), (**b**) **6** (**rBpyZ**) + 0.5 equiv. Zn^2+^, and (**c**) **6** (**rBpyZ**) + 1.0 equiv. Zn^2+^ in DMSO-*d*_6_.

We previously reported on the properties of the Zn^2+^-coordinated bipyridine moiety of **3** and its structural analogs using ^1^H-NMR [17]. As **6** (**rBpyZ**) exhibited a fluorescence response distinct from **3–5**, we investigated the Zn^2+^ coordination site of **6** (**rBpyZ**) using ^1^H-NMR spectroscopy. As shown in Figure 4, the protons of the 3- and 3′-positions of **6** (**rBpyZ**) shifted 0.04 or 0.9 ppm downfield when 1.0 equiv. of Zn^2+^ was added. The NH_2_ proton peak at 5.26 ppm moved to 6.29 ppm without any change in integral values. These results were similar to the NMR results of **3**, indicating that the Zn^2+^ coordinated with the 2,2′-bipyridine moiety of **6** (**rBpyZ**) and that amino-imino tautomerism did not occur. These results also suggested that the ratiometric property of **6** (**rBpyZ**) was not due to a change of the Zn^2+^ coordination site but was rather caused by the stabilization of the highest occupied molecular orbital (HOMO) energy via the introduction of an electron-donating group, similar to that observed in other ratiometric probes [20]. Ratiometric probes that emit at two distinct wavelengths are beneficial for the sensitive and selective detection of targets. Subsequently, evaluations including a metal selectivity experiment, a test of the influence of pH on fluorescence, and the analysis of the fluorescence imaging of cells, were performed on the ratiometric compound **6** (**rBpyZ**).

**Figure 5:**
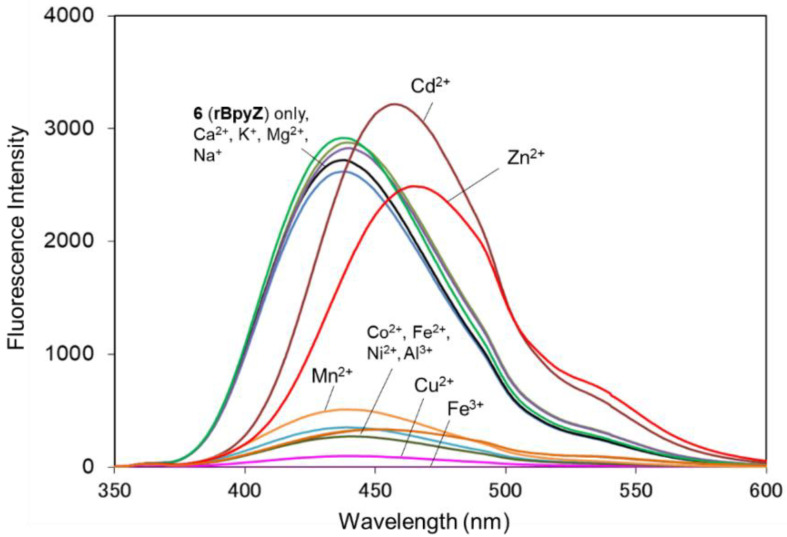
Fluorescence spectra of **6** (**rBpyZ**) (1 µM) in HEPES buffer (100 mM, 50% EtOH, pH = 7.4, excitation wavelength: 326 nm) upon the addition of different metal cations (100 µM).

**Figure 6:**
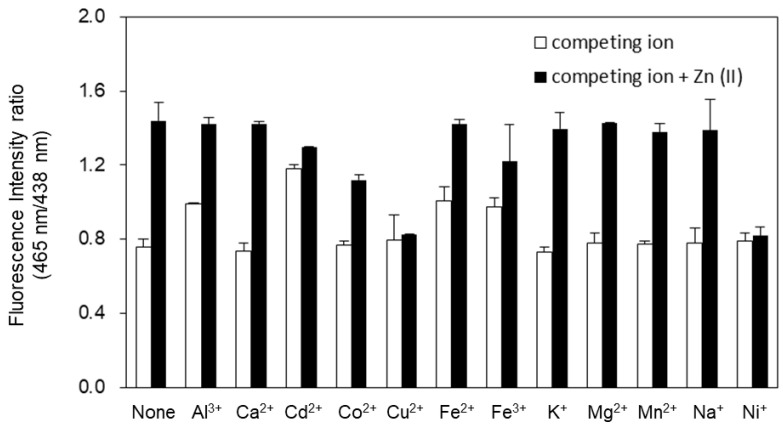
Competitive binding experiments of **6** (**rBpyZ**), in which competing metal ions (10^−3^ M) and Zn^2+^ (10^−3^ M) were added to **6** (**rBpyZ**) (10^−6^ M) in HEPES buffer (100 mM, 50% EtOH, pH = 7.4).

To investigate the selectivity of **6** (**rBpyZ**) toward cations, the fluorescence spectra of **6** (**rBpyZ**) in the presence of various cations (Al^3+^, Ca^2+^, Cd^2+^, Co^2+^, Cu^2+^, Fe^2+^, Fe^3+^, K^+^, Mg^2+^, Mn^2+^, Na^+^, Ni^2+^, and Zn^2+^) were recorded, and are displayed in Figure 5. We note that the addition of Cd^2+^ to **6** yields a similarly enhanced fluorescence spectrum to that produced with the addition of Zn^2+^. It has been reported that various Zn^2+^ fluorescence probes also detect Cd^2+^ owing to both ions being in the same column of the periodic table [12]. However, the concentration of free Cd^2+^ is very low in living systems and can therefore be assumed to have a negligible influence on cellular Zn^2+^ imaging. The addition of alkali ions (Na^+^; K^+^) and alkaline-earth ions (Ca^2+^; Mg^2+^), which exist ubiquitously at millimolar concentrations in living systems, cause no change to the spectrum of **6** (**rBpyZ**). In the transition metal cations (Co^2+^, Cu^2+^, Fe^2+^, Fe^3+^, Mn^2+^, Ni^2+^, and Al^3+^), fluorescence quenching and small bathochromic shifts in the emission wavelength were observed, indicating that these cations also form a complex with **6** (**rBpyZ**). However, binary competition experiments of **6** (**rBpyZ**) between Zn^2+^ and other cations, using the ratio of fluorescence intensity at 465 nm to that at 438 nm (F_46__5_/F_438_) (Figure 6), showed that in the presence of Co^2+^, Fe^2+^, Fe^3+^, Mn^2+^, and Al^3+^, **6** (**rBpyZ**) selectively detects Zn^2+^. **6** (**rBpyZ**) is not selective for Zn^2+^ when in the presence of either Cu^2+^ or Ni^2+^. Still, this lack of preference may have little impact on visualizing cellular Zn^2+^ as the cellular concentrations of free Cu^2+^ and Ni^2+^ are also very low. 

**Figure 7:**
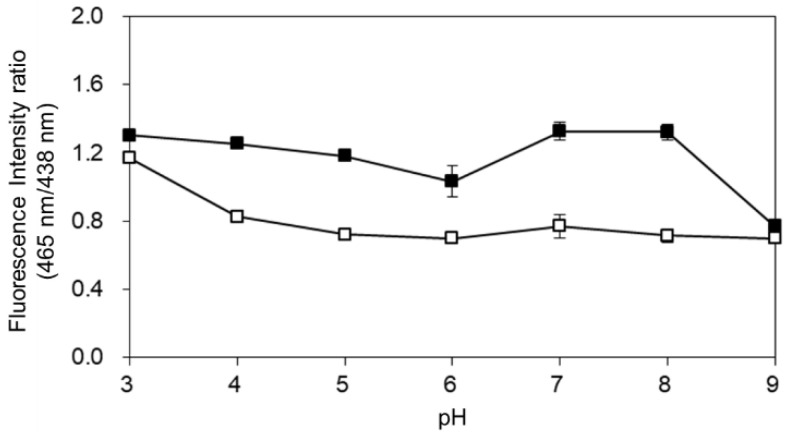
Effects of pH on the fluorescence properties of **6** (**rBpyZ**) in the absence (□) and presence (■) of Zn^2+^.

Figure 7 shows the effects of pH on the Zn^2+^ detection ability of **6** (**rBpyZ**). F_46__5_/F_438_ of **6** (**rBpyZ**) was measured in buffer solutions at different pH values in the absence and presence of Zn^2+^. Under both conditions, F_46__5_/F_438_ was approximately constant between pH 5.0–8.0. In addition, a limit of detection (LOD) of 0.10 nM at pH 7.0 buffer solution was calculated using the equation LOD = 3σ/slope. These results indicated that **6** (**rBpyZ**) could function as a sensitive Zn^2+^ probe under physiological conditions. We subsequently explored its usefulness in cellular applications, which we report in the next section.

### 2.3. Zn^2+^ Cellular Imaging

**Figure 8:**
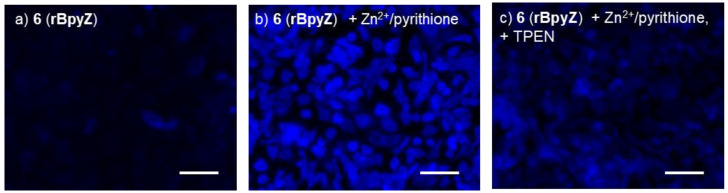
Fluorescence images of exogenously added Zn^2+^ in A549 cells: (**a**) fluorescence image of cells with **6** (**rBpyZ**) (100 μM); (**b**) fluorescence image of cells incubated with **6** (**rBpyZ**) (100 μM) in the presence of Zn^2+^/pyrithione (zinc ionophore; 100 μM); (**c**) fluorescence image of cells in (**b**) after treatment with TPEN (100 μM). Scale bars = 300 μm.

Figure 8 shows fluorescence microscopy images of human lung adenocarcinoma cells (A549). The cells were exposed to 100 µM **6** (**rBpyZ**) for 30 min at 37 °C, which produced very weak fluorescence (Figure 8a). In contrast, the cells incubated with 100 µM **6** (**rBpyZ**) for 30 min and 100 µM Zn^2+^ for another 30 min exhibited a bright fluorescence signal in the cells (Figure 8b). The fluorescence signal was reduced by treatment with a cell membrane permeable Zn^2+^ chelator, *N*,*N*,*N*’,*N*’-tetrakis(2-pyridylmethyl)ethylenediamine (TPEN) [21], indicating that **6** (**rBpyZ**) exhibited fluorescence in response to intracellular Zn^2+^ (Figure 8c). In addition, no significant cytotoxicity was observed during this study.

**Figure 9:**
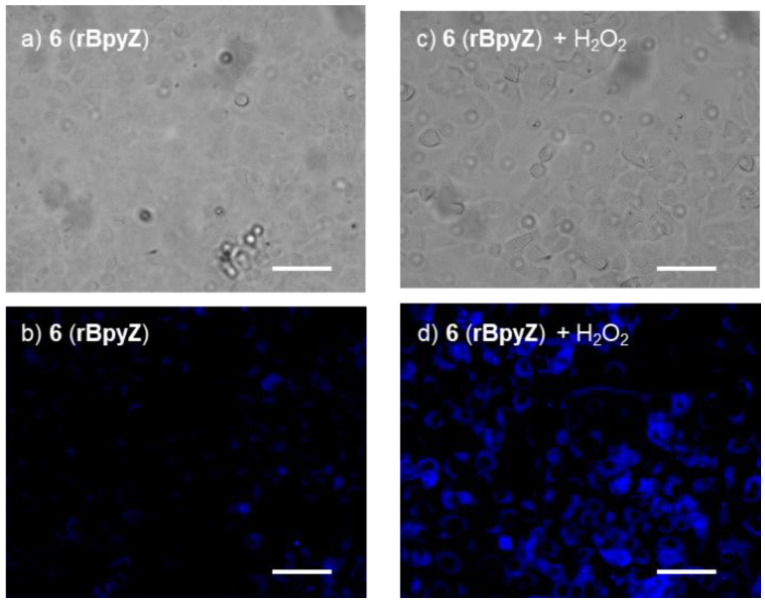
Fluorescence images of endogenous Zn^2+^ in A549 cells: (**a**) bright-field image of cells with **6** (**rBpyZ**) (100 μM); (**b**) fluorescence image of cells with **6** (**rBpyZ**) (100 μM); (**c**) bright field image incubated with **6** (**rBpyZ**) (100 μM) after incubation with H_2_O_2_ (100 μM) for 24 h; (**d**) fluorescence image incubated with **6** (**rBpyZ**) (100 μM) after incubation with H_2_O_2_ (100 μM) for 24 h. Scale bars = 300 μm.

Therefore, we further investigated the possibility of detecting endogenous Zn^2+^ in the apoptotic cells. Zn^2+^ is known as a regulator of apoptosis and is released from intracellular stores during apoptosis [22]. As shown in Figure 9, in the H_2_O_2_-induced apoptotic cells, a bright fluorescence signal was observed after incubation with 100 µM **6** (**rBpyZ**). It was confirmed that this observed fluorescence signal does not originate from the direct effect of H_2_O_2_ on **6** (**rBpyZ**) (Figure S3). These data indicated that ratiometric probe **6** (**rBpyZ**) could detect endogenous Zn^2+^.

## 3. Materials and Methods

### 3.1. Materials and Instruments

All reagents and chemicals were of the highest grade available. ^1^H and ^13^C-NMR spectra were recorded on JEOL ECP-400 NMR and Varian Gemini 300 systems at room temperature. The chemical shifts are reported in parts per million (ppm) relative to tetramethylsilane (TMS) as the reference. Mass spectra (MS) and high-resolution mass spectrometry (HRMS) were performed using a JMS-700 spectrometer (JEOL, Tokyo, Japan). UV/Vis absorption spectra were acquired with a UV/Vis spectrophotometer (UV-2450, Shimadzu, Kyoto, Japan). Fluorescence spectra were acquired using a fluorescence spectrophotometer (FP-8300ST, Jasco, Tokyo, Japan).

### 3.2. Synthesis of 4-(Methylsulfanyl)-5-Phenyl-[2,2′-Bipyridin]-6-Amine (**3**)

To a solution of 3,3-bis(methylsulfanyl)-2-(phenylsulfonyl)acrylonitrile (**1**) (560 mg, 2.5 mmol) and 2-phenylacetonitrile (**2a**) (439 mg, 3.8 mmol) in DMSO (20 mL), powdered sodium hydroxide (200 mg, 5.0 mmol) was added, and the mixture was stirred for 2 h at room temperature. The reaction solution was poured into 200 mL of ice water and neutralized with a 10% hydrochloric acid solution. The mixture was extracted with chloroform (3 × 50 mL), washed with brine (50 mL), and dried over anhydrous sodium sulfate. After concentration under reduced pressure conditions, a mixture of the residue and 150 mL of 28% aqueous ammonia was refluxed for 1 h. After the evaporation of the ammonia and water, the residue was chromatographed on a silica gel column using toluene as an eluent to obtain 205 mg (0.7 mmol, 28%) of **3** as colorless needles. Mp: 119–120 °C. ^1^H-NMR (DMSO-*d*_6_, 400 MHz) δ 2.50 (s, 3H), 5.26 (brs, 2H), 7.27 (d, *J* = 7.3 Hz, 2H), 7.41 (m, 2H), 7.50 (dd, *J* = 7.3, 7.8 Hz, 2H), 7.70 (s, 1H), 7.91 (dd, *J* = 7.8, 7.8 Hz, 1H), 8.30 (d, *J* = 7.8 Hz, 1H), 8.67 (d, *J* = 4.4 Hz, 1H). ^13^C-NMR (DMSO-*d*_6_, 100 MHz) δ 13.9, 104.8, 117.5, 120.4, 123.9, 128.4, 129.3, 129.9, 129.9, 135.3, 137.0, 149.0, 149.7, 152.4, 155.4, 155.8. MS *m*/*z*: 294 [M + H^+^]. HRMS Calcd for C_17_H_16_N_3_S [M + H^+^]: 294.1065. Found: 294.1040.

### 3.3. Synthesis of 5-(4-Chlorophenyl)-4-(Methylsulfanyl)-[2,2′-Bipyridin]-6-Amine (**4**)

Compound **4** was prepared from **1** (565 mg, 2.5 mmol) and 2-(4-chlorophenyl)acetonitrile (**2b**) (574 mg, 3.8 mmol) in a manner similar to that described for the synthesis of **3**. The residue was chromatographed on a silica gel column using toluene as an eluent to obtain 121 mg (0.4 mmol, 15%) of **4** as colorless needles. Mp: 243–244 °C. ^1^H-NMR (DMSO-*d*_6_, 400 MHz) δ 2.50 (s, 3H), 5.41 (brs, 2H), 7.27 (d, *J* = 8.4 Hz, 2H), 7.47–7.49 (m, 2H), 7.49 (dd, *J* = 8.4 Hz, 2H), 7.65 (s, 1H), 7.99 (dd, *J* = 6.8, 8.0 Hz, 1H), 8.26 (d, *J* = 8.4 Hz, 1H), 8.74 (d, *J* = 4.4 Hz, 1H). ^13^C-NMR (DMSO-*d*_6_, 100 MHz) δ 14.8, 120.4, 121.1, 124.9, 128.1, 129.3, 132.0, 132.1, 132.2, 133.9, 137.0, 137.7, 149.5, 151.9, 152.7, 159.4. MS *m*/*z*: 328 [M + H^+^]. HRMS Calcd for C_17_H_15_ClN_3_S [M + H^+^]: 328.0675. Found: 328.0610.

### 3.4. Synthesis of 4-(Methylsulfanyl)-5-(4-(Trifluoromethyl)Phenyl)-[2,2′-Bipyridin]-6-Amine (**5**)

Compound **5** was prepared from **1** (565 mg, 2.5 mmol) and 2-(4-(trifluoromethyl)phenyl)acetonitrile (**2c**) (693 mg, 3.8 mmol) in a manner similar to that described for the synthesis of **3**. The residue was chromatographed on a silica gel column using toluene as an eluent to obtain 42 mg (0.1 mmol, 5%) of **5** as colorless needles. Mp: 228–229 °C. ^1^H-NMR (CDCl_3_, 300 MHz) δ 2.49 (s, 3H), 6.89 (brs, 2H), 7.37–7.42 (m, 2H), 7.53 (d, *J* = 7.84 Hz, 2H), 7.69 (d, *J* = 7.8 Hz, 2H), 7.79 (m, 3H), 7.91 (d, *J* = 8.4 Hz, 1H), 8.66 (d, *J* = 5.4 Hz, 1H). ^13^C-NMR (DMSO-*d*_6_, 75 MHz) δ 15.5, 100.2, 119.9, 122.6, 125.1, 125.5, 125.6, 126.2, 130.0, 130.4, 130.9, 137.6, 138.6, 140.7, 147.8, 149.7, 152.8, 160.1. MS *m*/*z*: 362 [M + H^+^]. HRMS Calcd for C_18_H_15_F_3_N_3_S [M + H^+^]: 362.0938. Found: 362.0902.

### 3.5. Synthesis of 5-(4-Methoxyphenyl)-4-(Methylsulfanyl)-[2,2′-Bipyridin]-6-Amine (**6**, **rBpyZ**) 

Compound **6** (**rBpyZ**) was prepared from **1** (565 mg, 2.5 mmol) and 2-(4-2-(4-methoxyphenyl)acetonitrile (**2d**) (551 mg, 3.8 mmol) in a manner similar to that described for the synthesis of **3**. The residue was chromatographed on a silica gel column using toluene as an eluent to obtain 364 mg (1.1 mmol, 44%) of **6** (**rBpyZ**) as colorless needles. Mp 233–234 °C. ^1^H-NMR (CDCl_3_, 300 MHz) δ 2.45 (s, 3H), 3.84 (s, 3H), 4.35 (brs, 2H), 7.02 (d, *J* = 8.7 Hz, 2H), 7.25–7.30 (m, 1H), 7.34 (d, *J* = 8.7 Hz, 2H), 7.72 (s, 1H), 7.76–7.85 (m, 1H), 8.30 (d, *J* = 8.1 Hz, 1H), 8.65 (m, 1H). ^13^C-NMR (CDCl_3_, 75 MHz) δ 14.9, 55.5, 100.0, 107.0, 114.1, 115.1, 121.3, 123.6, 127.5, 131.4, 131.5, 149.2, 151.4, 153.4, 155.7, 156.5, 159.8. MS *m*/*z*: 324 [M + H^+^]. HRMS calcd for C_18_H_17_N_3_OS [M + H^+^]: 324.1170. Found: 324.1195.

### 3.6. Spectroscopic Studies

The solutions of perchlorate salts of metal ions (Na^+^, K^+^, Mg^2+^, Ca^2+^, Fe^2+^, Fe^3+^, Ni^2+^, Zn^2+^, Cd^2+^, Co^2+^, Cu^2+^, Mn^2+^, and Al^3+^) were prepared by dissolving them in deionized water. To prepare compound stock solutions (1 × 10^−2^ M), each compound was dissolved in DMSO. The UV/Vis and fluorescence analysis of each compound was performed in the absence and presence of metal ions in EtOH/H_2_O (1:1, *v*/*v*). Job plot experiments were performed to determine the complex stoichiometry of **4–6** (**rBpyZ**) to Zn^2+^. The dissociation constants (*K*_d_) were determined using a 0–9 mM ZnSO_4_/10 mM nitrilotriacetic acid (NTA) system and Benesi–Hildebrand plots [23,24,25]. The metal ion selectivity of **6** (**rBpyZ**) was investigated using 10^−2^ M metal cation solutions. The effects of pH change on the fluorescence of **6** (**rBpyZ**) were evaluated using various buffers: citrate buffer (pH 3.0–6.0), HEPES buffer (pH 7.0–8.0), and tris-hydrochloric acid buffer (pH 9.0).

### 3.7. Microscope Imaging of Zinc Fluorescence in Cells

The cell line A549 human lung adenocarcinoma from RIKEN BRC (Ibaraki, Japan) was cultured in Dulbecco’s modified Eagle’s medium (DMEM) supplemented with 10% fetal bovine serum and 1% penicillin. Cells were incubated at 37 °C in a humidified atmosphere containing 5% CO_2_. To evaluate the cell membrane permeability of **6** (**rBpyZ**), the cells were incubated with **6** (**rBpyZ**) (100 μM) for 30 min at 37 °C, washed with phosphate-buffered saline (PBS), and incubated with Zn^2+^/pyrithione (100 μM) for 30 min at 37 °C. For TPEN studies, the cells were incubated with TPEN (100 μM) for an additional 30 min at 37 °C after incubation with Zn^2+^/pyrithione (100 μM). To detect endogenous Zn^2+^ in A549 cells, the cells were incubated with hydrogen peroxide (100 µM) in DMEM for 24 h at 37 °C. After washing with PBS, the treated cells were incubated with **6** (**rBpyZ**) (100 μM) for 30 min at 37 °C. The fluorescence images of the incubated cells were acquired using a fluorescence microscope BZ-X710 (Keyence, Osaka, Japan). 

## 4. Conclusions

To reduce the influence of background fluorescence and achieve the sensitive detection of endogenous Zn^2+^ in cells, we designed and synthesized 6-amino-2,2′-bipyridine-based small molecular weight Zn^2+^ fluorescence compounds with electron-withdrawing or electron-donating groups on the allyl group at position 5 of the central pyridine ring. Among the synthesized compounds, compound **6** (**rBpyZ**) bearing a methoxy group exhibited ratiometric fluorescence profiles at 438 nm and 465 nm in the absence or presence of Zn^2+^. In addition, **6** (**rBpyZ**) showed a high Zn^2+^ binding affinity (*K*_d_ = 0.77 nM), a large Stokes shift (over 139 nm), high Zn^2+^ selectivity, and high stability under physiological pH conditions, making it useful as a fluorescence Zn^2+^ probe. Fluorescence microscopy studies indicated that **6** (**rBpyZ**) possessed cell membrane permeability in living human lung adenocarcinoma cells and enabled the visualization of endogenous labile Zn^2+^ in the cells during apoptosis. We expect that **6** (**rBpyZ**) will be a valuable and efficient probe for elucidating the biological functions of Zn^2+^.

## Figures and Tables

**Table 1:** Fluorescence data for compounds **3**–**6** (**rBpyZ**).

Compounds	λ max	log є	Ex max	Em max	SS ^a^	Φ ^b^	*K*_d_ (nM)
	(nm)		(nm)	(nm)	(nm)		
**3**	352	4.15	359	466	107	0.07 (−Zn^2+^)	2.2
						0.37 (+Zn^2+^)	
**4**	350	3.98	360	467	107	0.05 (−Zn^2+^)	1.2
						0.35 (+Zn^2+^)	
**5**	350	3.98	359	465	106	0.05 (−Zn^2+^)	3
						0.52 (+Zn^2+^)	
**6 (rBpyZ)**	319	4.19	326	437 (−Zn^2+^)	111 (−Zn^2+^)	0.45 (−Zn^2+^)	0.77
				465 (+Zn^2+^)	139 (+Zn^2+^)	0.63 (+Zn^2+^)	

^a^ Stokes shift. ^b^ The fluorescence quantum yield values were measured with respect to quinine sulfated solution as standard.

## Data Availability

The data presented in this study are available in the present article and Supplementary Files.

## Supplementary Materials

The following are available online: Figure S1: Job’ plot analysis of **4**, **5,** and **6** (**rBpyZ**); Figure S2: Benesi–Hildebrand analysis of **4**, **5,** and **6** (**rBpyZ**); Figure S3: Fluorescence spectra of **6** (**rBpyZ**) upon the addition of H_2_O_2_. ^1^H- and ^13^C-NMR spectra are available online.

## Sample Availability

Samples of the compounds are available from the authors.
