# Supplementary material for: High-Affinity Ratiometric Fluorescence Probe Based on 6-Amino-2,2′-Bipyridine Scaffold for Endogenous Zn2+ and Its Application to Living Cells
Source: Molecules. 2022 Feb 14;27(4):1287. doi: 10.3390/molecules27041287 (PMC8876810; doi:10.3390/molecules27041287)
Supplement: Supplementary file 1 [file molecules-27-01287-s001.zip › molecules-1573685-supplementary.pdf]

Supplementary Materials

High-affinity ratiometric fluorescence probe based on 6-amino-2,2'-bipyridine scaffold for endogenous Zn^{2+} and its application to living cells

Masayori Hagimori[1] • Fumiko Hara[1] • Naoko Mizuyama[2] • Takeshi Fujino[3] • Hideo Saji[4] • Takahiro Mukai[5]

[1]Laboratory of Analytical Chemistry, Faculty of Pharmaceutical Sciences, Mukogawa Women's University, 11-68 Koshien Kyubancho, Nishinomiya 663-8179, Japan

[2]Division of Medical Innovation, Translational Research Center for Medical Innovation, 1-5-4 Minatojima-minamimachi, Chuo-ku, Kobe 650-0047, Japan

[3]Graduate School of Science and Engineering, Saitama University, 255 Shimo-Okubo, Sakura-ku, Saitama 338-8570, Japan

[4]Graduate School of Pharmaceutical Sciences, Kyoto University, 46–29 Yoshida-Shimoadachi-cho, Sakyo-ku, Kyoto 606-8501, Japan

[5] Laboratory of Biophysical Chemistry, Kobe Pharmaceutical University, 4-19-1 Motoyamakita Machi, Higashinada-Ku, Kobe 658-8558

Contents

1. Figure S1. Job' plot analysis of **4**, **5** and **6** (**rBpyZ**)

2. Figure S2. Benesi–Hildebrand analysis of **4**, **5** and **6** (**rBpyZ**)

3. Figure S3. Fluorescence spectra of **6** (**rBpyZ**) upon the addition of H_2O_2

4. Figure S4. ^{1}H NMR spectrum of **4**

5. Figure S5. ^{13}C NMR spectrum of **4**

6. Figure S6. ^{1}H NMR spectrum of **5**

7. Figure S7. ^{13}C NMR spectrum of **5**

8. Figure S8. ^{1}H NMR spectrum of **6** (**rBpyZ**)

9. Figure S9. ^{13}C NMR spectrum of **6** (**rBpyZ**)

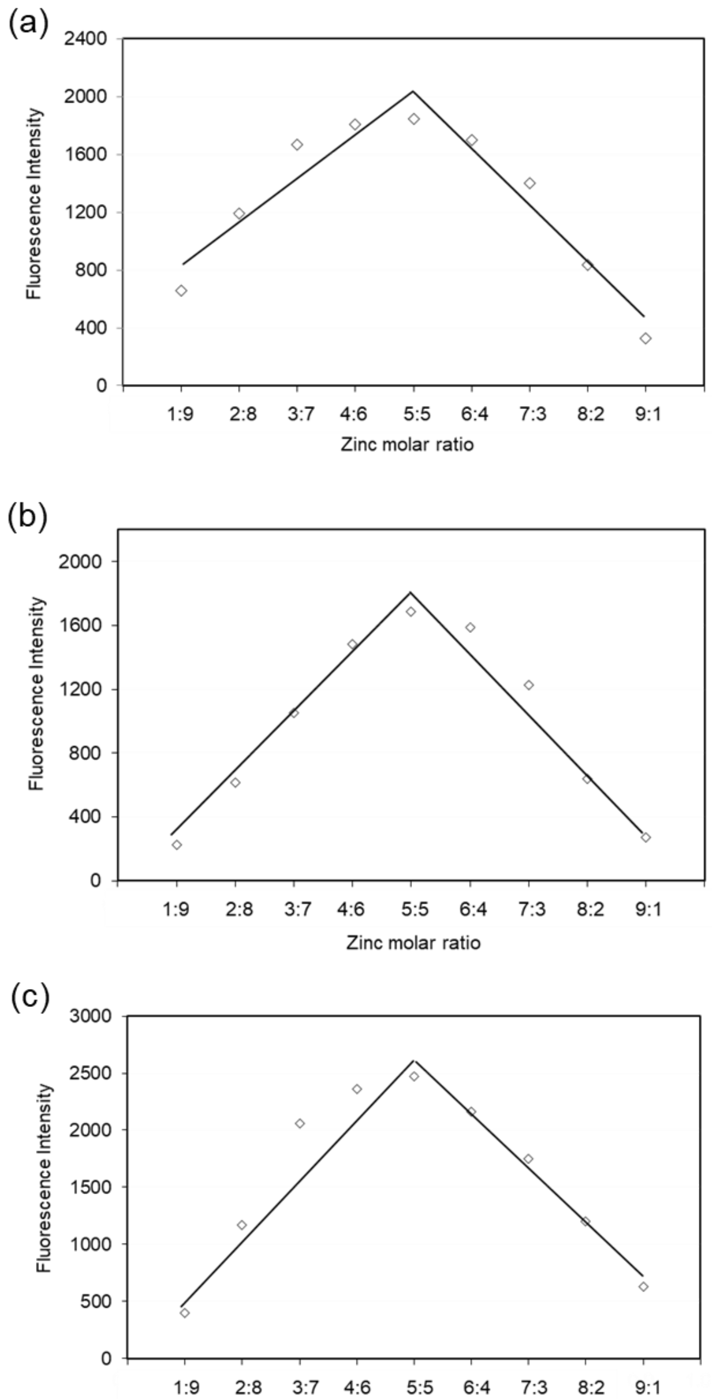

Figure S1. Job' plot analysis of (a) **4**, (b) **5** and (c) **6** (**rBpyZ**).

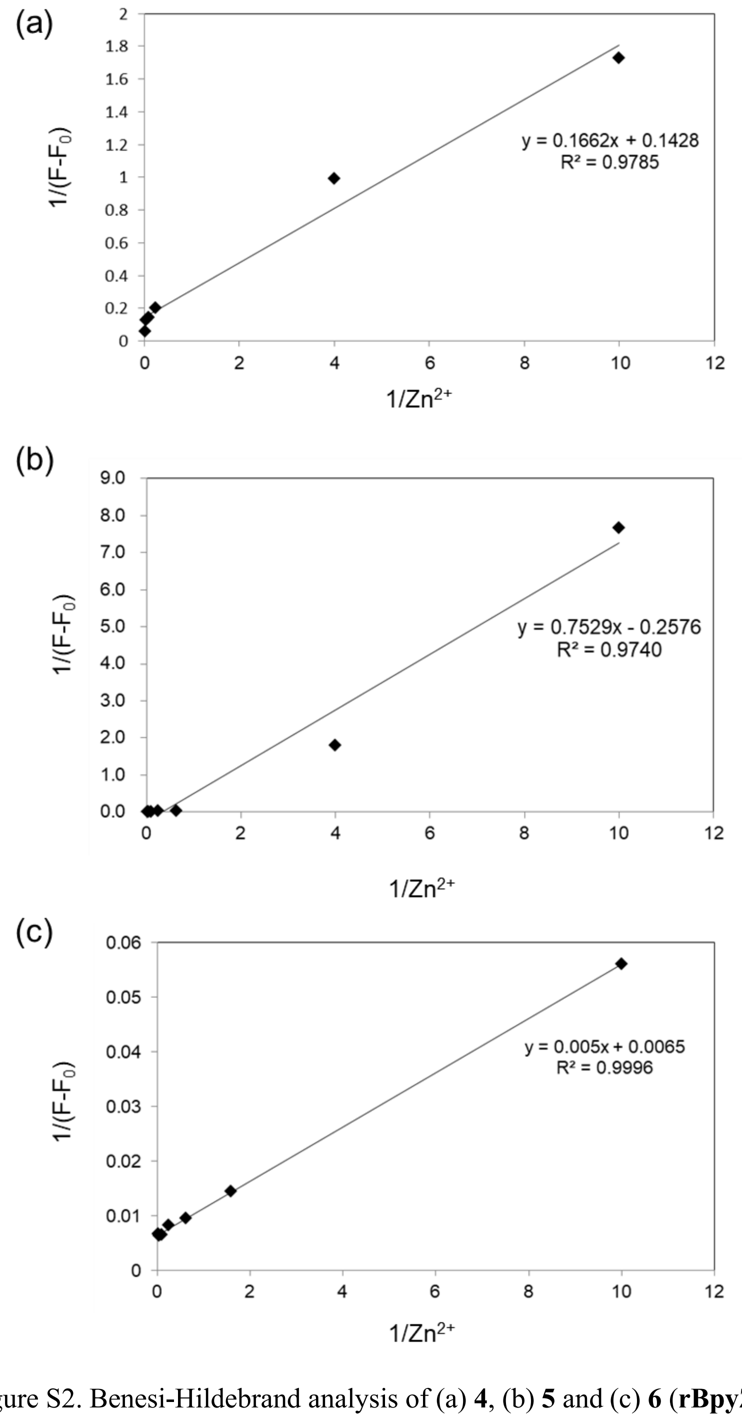

Figure S2. Benesi-Hildebrand analysis of (a) **4**, (b) **5** and (c) **6** (**rBpyZ**).

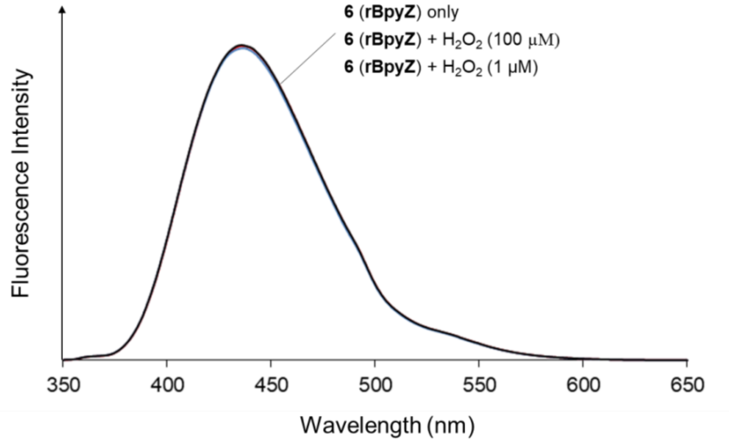

Figure S3. Fluorescence spectra of **6** (**rBpyZ**) (1 μM) upon the addition of H_2O_2 in HEPES buffer (100 mM, 50% EtOH, pH = 7.4, excitation wavelength: 326 nm).

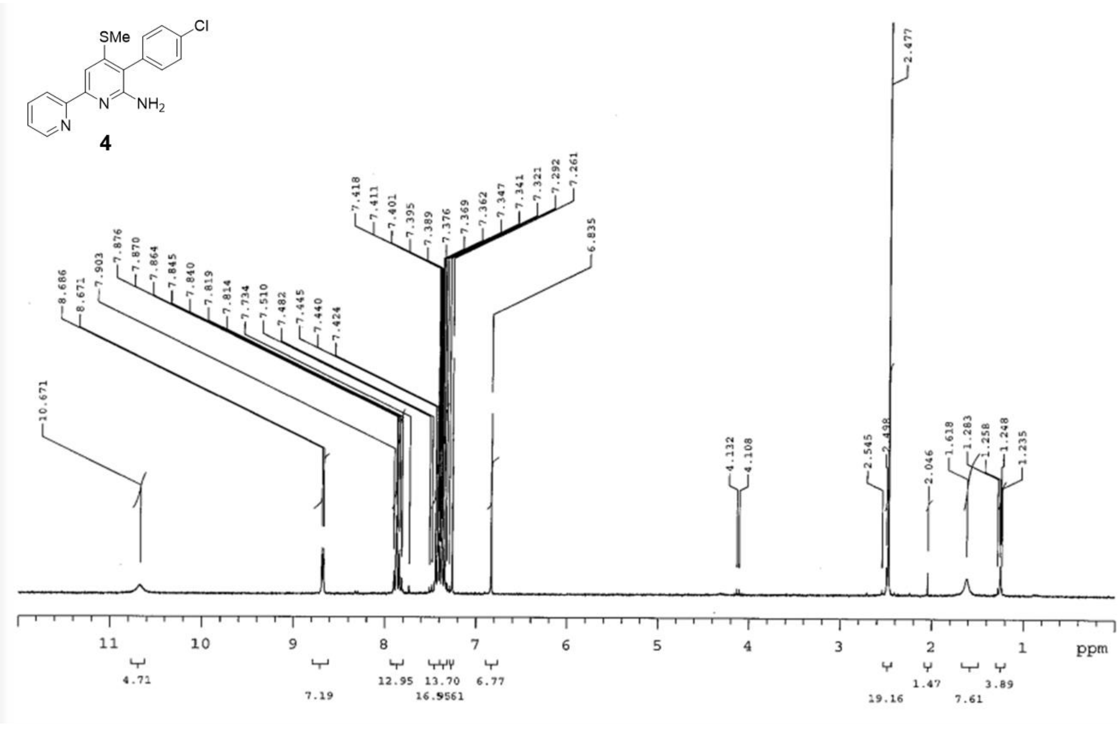

Figure S4. ^{1}H NMR spectrum of **4**

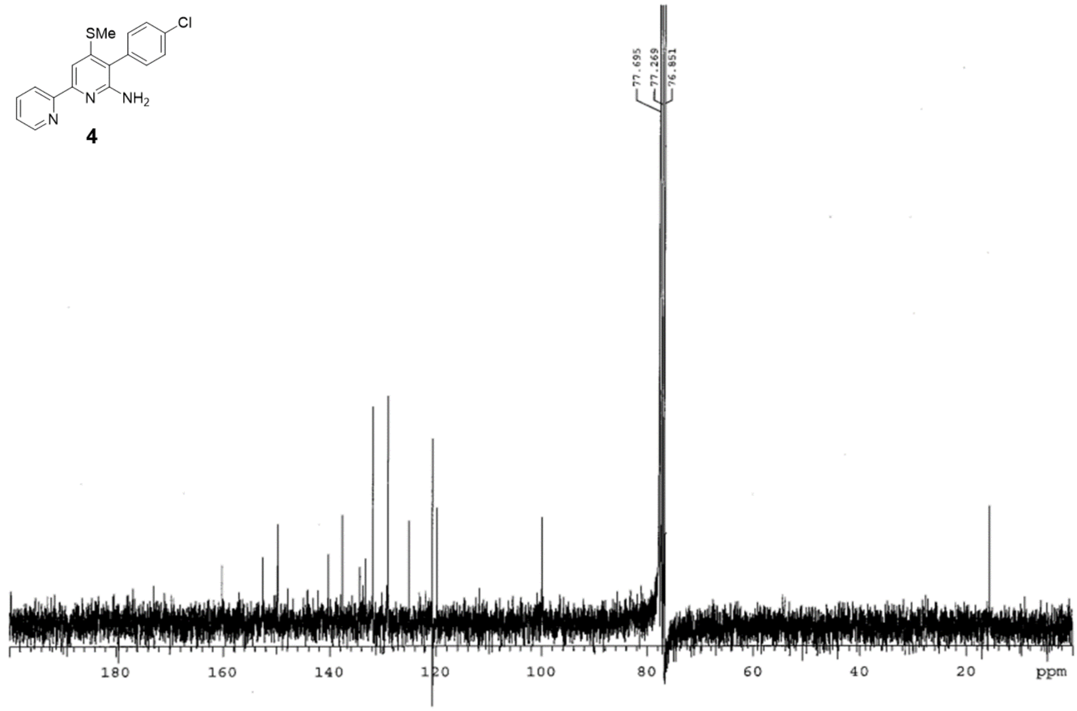

Figure S5. ^{13}C NMR spectrum of 4

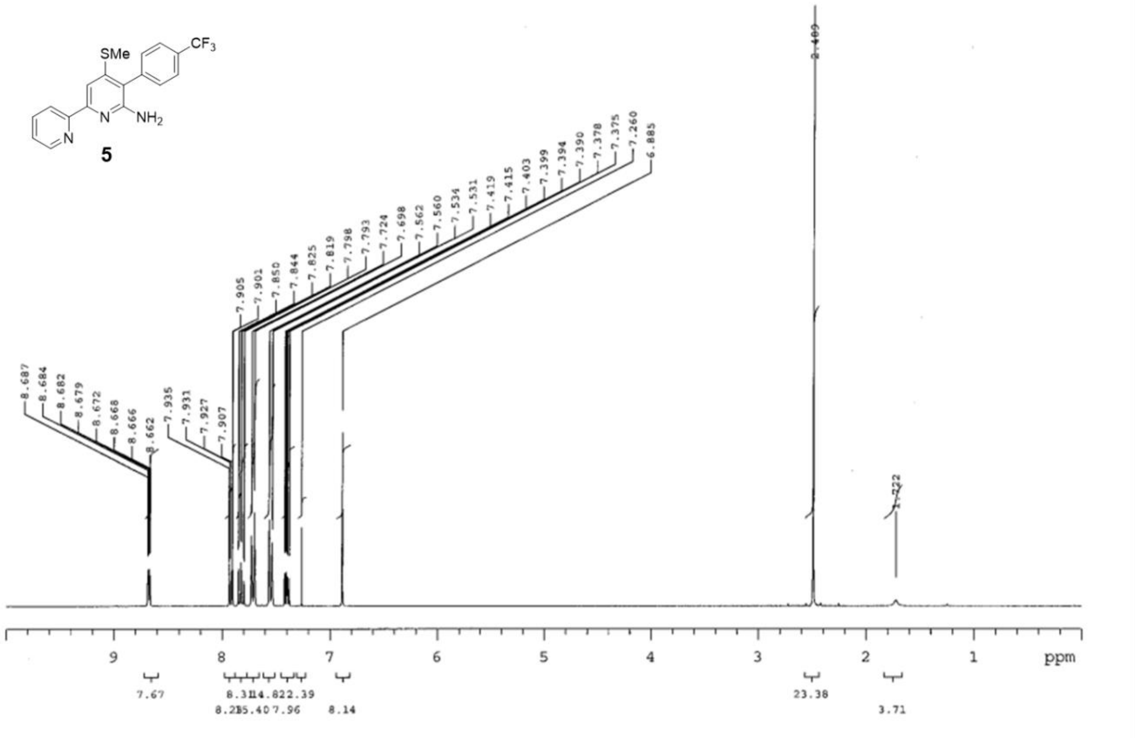

Figure S6. ^{1}H NMR spectrum of **5**

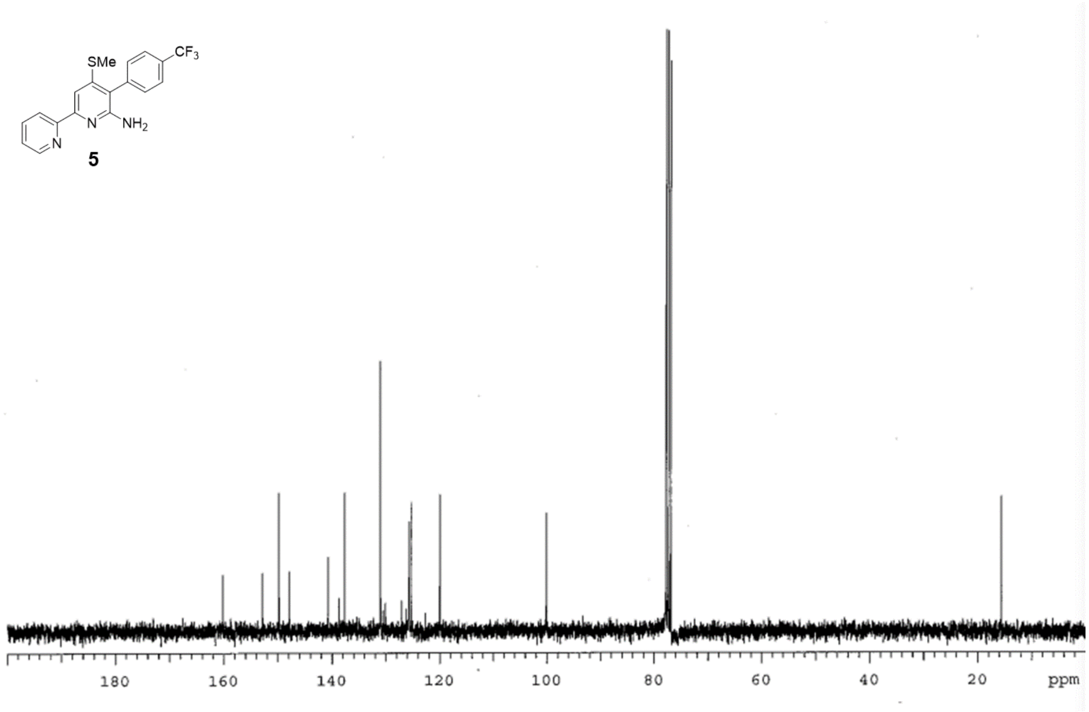

Figure S7. ^{13}C NMR spectrum of **5**

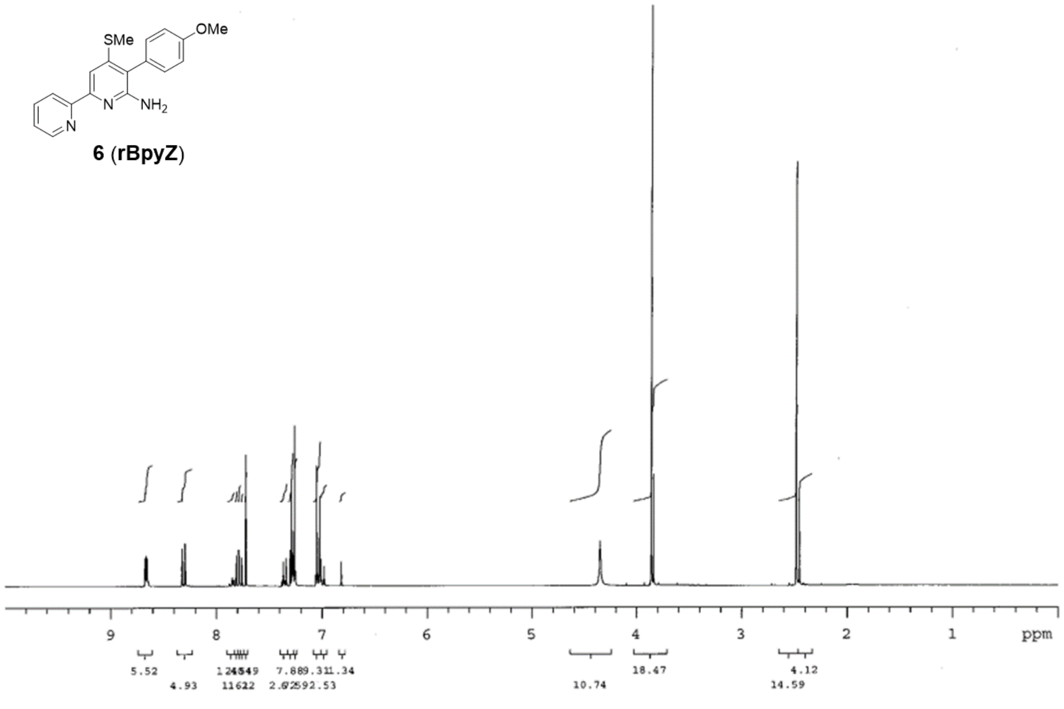

Figure S8. ^{1}H NMR spectrum of **6** (**rBpyZ**)

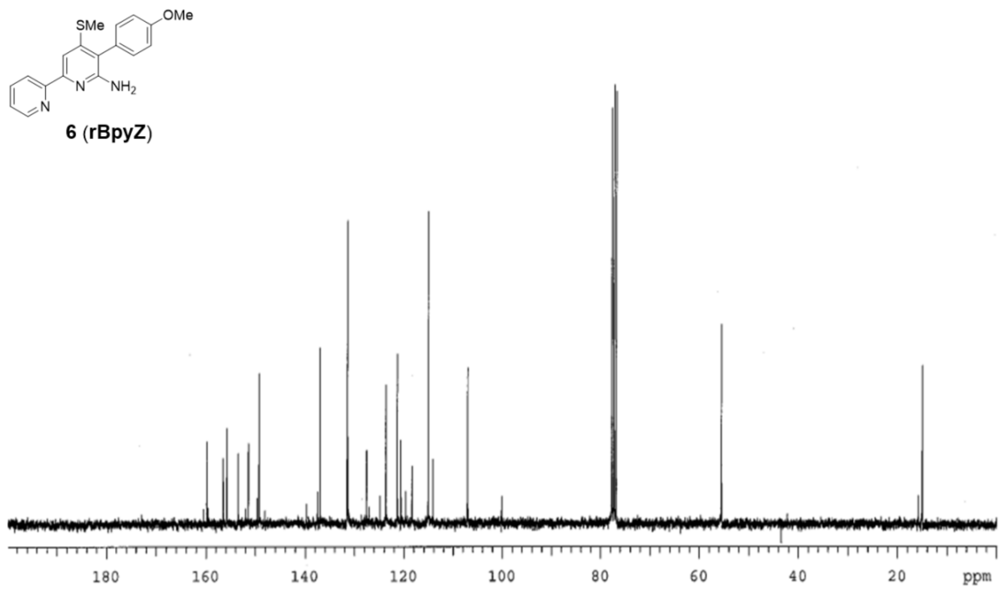

Figure S9. ^{13}C NMR spectrum of **6** (**rBpyZ**)
